# Generation of recombinant single-chain antibodies neutralizing the cytolytic activity of vaginolysin, the main virulence factor of *Gardnerella vaginalis*

**DOI:** 10.1186/1472-6750-11-100

**Published:** 2011-11-03

**Authors:** Milda Pleckaityte, Edita Mistiniene, Rita Lasickiene, Gintautas Zvirblis, Aurelija Zvirbliene

**Affiliations:** 1 Institute of Biotechnology, Vilnius University, Graiciuno 8, LT-02241, Vilnius, Lithuania

## Abstract

**Background:**

*Gardnerella vaginalis *is identified as the predominant colonist of the vaginal tract in women with bacterial vaginosis. Vaginolysin (VLY) is a protein toxin released by *G. vaginalis*. VLY possesses cytolytic activity and is considered as a main virulence factor of *G. vaginalis*. Inhibition of VLY-mediated cell lysis by antibodies may have important physiological relevance.

**Results:**

Single-chain variable fragments of immunoglobulins (scFvs) were cloned from two hybridoma cell lines producing neutralizing antibodies against VLY and expressed as active proteins in *E. coli*. For each hybridoma, two variants of anti-VLY scFv consisting of either VL-VH or VH-VL linked with a 20 aa-long linker sequence (G_4_S)_4 _were constructed. Recovery of scFvs from inclusion bodies with subsequent purification by metal-chelate chromatography resulted in VLY-binding proteins that were predominantly monomeric. The antigen-binding activity of purified scFvs was verified by an indirect ELISA. The neutralizing activity was investigated by *in vitro *hemolytic assay and cytolytic assay using HeLa cell line. Calculated apparent K_d _values and neutralizing potency of scFvs were in agreement with those of parental full-length antibodies. VH-VL and VL-VH variants of scFvs showed similar affinity and neutralizing potency. The anti-VLY scFvs derived from hybridoma clone 9B4 exhibited high VLY-neutralizing activity both on human erythrocytes and cervical epithelial HeLa cells.

**Conclusions:**

Hybridoma-derived scFvs with VLY-binding activity were expressed in *E. coli*. Recombinant anti-VLY scFvs inhibited VLY-mediated cell lysis. The monovalent scFvs showed reduced affinity and neutralizing potency as compared to the respective full-length antibodies. The loss of avidity could be restored by generating scFv constructs with multivalent binding properties. Generated scFvs is the first example of recombinant single-chain antibodies with VLY-neutralizing activity produced in prokaryote expression system. *G. vaginalis *caused infections continue to be a world-wide problem, therefore neutralizing recombinant antibodies may provide novel therapeutic agents useful in the treatment of bacterial vaginosis and other diseases caused by *G. vaginalis*.

## Background

*Gardnerella vaginalis *is a facultative anaerobic bacterium of the *Bifidobacteriaceae *family and the sole member of the genus *Gardnerella *[1]. *G. vaginalis *is the predominant microorganism of the vaginal tract in women with bacterial vaginosis (BV) [2,3]. BV is highly prevalent, affecting almost one third of women [4]. Being an important medical condition itself, BV is associated with several serious adverse outcomes including preterm birth and infertility [2,5], endometritis [6], and acquisition of other sexually transmitted infections [7]. Moreover, *G. vaginalis *has been linked with infections outside the reproductive system. It has been demonstrated that *G. vaginalis *may cause urinary tract infections in men [8], retinal vasculitis [9], acute hip arthritis in a renal transplant recipients [10], vertebral osteomyelitis [11] and bacteremia in a previously healthy man [12]. These data indicate that *G. vaginalis *may be more virulent than previously expected. It was demonstrated that certain strains of *G. vaginalis *are able to form biofilms [3,13]. The genomic analysis support findings on *G. vaginalis *virulence features such as its ability to adhere to vaginal epithelium, biofilm formation, cytotoxic activity and also provides other features important to the role of *G. vaginalis *in BV development [14,15].

The main virulence factor of *G. vaginalis *is the protein toxin vaginolysin (VLY) [16,17]. The VLY belongs to the cholesterol-dependent cytolysins (CDCs), a family of pore-forming toxins [18]. These toxins disrupt plasma membranes causing cell lysis and are thought to play a key role in the virulence of bacteria [18]. VLY is a toxin specific to human cells as it recognizes the complement regulatory molecule CD59 [17,19,20]. Taken together the virulence properties of *G. vaginalis *allow the bacteria to adhere to the vaginal epithelium, produce a biofilm and secrete VLY that leads to cytolysis and tissue destruction [3].

The high recurrence rate of BV after antibiotic treatment or persistent BV over time [21,22] may prompt the development and use of recombinant antibodies as novel therapeutic agents for disease treatment. The effectiveness of neutralizing recombinant antibodies against other bacterial toxins, such as pneumolysin, Shiga toxin, *Clostridium difficile *toxin A, Salmonella SpvB toxin, heat-labile toxin from enterotoxigenic *E. coli*, botulinum neurotoxin has been demonstrated in previous studies [23-29]. Recombinant antibodies neutralizing the cytolytic activity of VLY have not yet been described.

Recently, we have developed a panel of monoclonal antibodies (MAbs) against VLY and demonstrated the ability of some MAbs to prevent the lysis of human erythrocytes *in vitro *[30].

In the current study, the hybridomas producing well-characterized MAbs 9B4 and 23A2 with the most potent VLY neutralizing activity were selected to construct recombinant single-chain variable fragments of immunoglobulins (scFvs). The scFvs were produced in *E. coli*, purified and characterized in comparison with the full-length parental MAbs. The ability of scFv to inhibit cytolytic activity of VLY *in vitro *has been demonstrated. Construction of recombinant scFv with a potent VLY-neutralizing activity may be considered as a first step in developing novel immunotherapeutic tools for the treatment of BV and other diseases caused by *G. vaginalis*.

## Methods

### Cloning and expression of anti-VLY scFvs

Total mRNA was isolated from 3 × 10^6 ^hybridoma cells producing neutralizing MAbs 9B4 and 23A2 [30]. Hybridoma cells were lysed in solution containing guanidine thiocyanate and extracted with phenol-chloroform at reduced pH [31]. The first strand of cDNA was prepared using RevertAid™ H Minus First Strand cDNA Synthesis Kit (Thermo Scientific Fermentas, Vilnius, Lithuania) according to manufacturer's instructions. The cDNA corresponding to the variable region of mouse IgG was obtained using sets of specific primers described previously [32]: 1) Mouse heavy chain constant region primer: 5'-TTAATAGACAGATGGGGGTGTCGTTTTGGC and mouse heavy chain FR1 region high degenerate primer 5'-CATATGSARGTNMAGCTGSAGSAGTC; 2) Mouse kappa chain constant region primer: 5'-TTAGGATACAGTTGGTGCAGCATC and mouse kappa chain FR1 region universal degenerated primer: 5'-CATATGGAYATTGTGMTSACMCARWCTMCA. For each chain, 5 separate clones were selected. Cloned DNA fragments were sequenced and found to be identical in all 5 clones. In a second PCR round, additional primers designed to introduce the 20 amino acid (aa)-long linker sequence (G_4_S)_4 _and NdeI/XhoI restriction sites were used. The VL and VH DNA fragments were fused in different orientation to result constructions VL-(G_4_S)_4_-VH and VH-(G_4_S)_4_-VL, respectively. The polymerase chain reactions (PCR) were carried out under standard conditions using High Fidelity PCR Enzyme Mix (Thermo Scientific Fermentas). The resulted DNA fragments encoding anti-VLY scFv were introduced by ligation into expression vector pET28a(+) (Merck, Darmstadt, Germany) digested with restriction endonucleases NdeI and XhoI (Thermo Scientific Fermentas). The expression plasmids were transfected into *E. coli *BL21(DE3) strain. The anti-VLY scFv synthesis was induced with 0.5 mM IPTG (isopropyl-β-D-thiogalactopyranoside) (Thermo Scientific Fermentas). After induction, the cell pellet was disrupted by sonication and centrifuged. The supernatant (soluble fraction) and the cell pellet (insoluble fraction) were then analyzed by 12.5% polyacrylamide gel electrophoresis (SDS-PAGE) under reducing conditions.

### Purification of anti-VLY scFvs

*E.coli *biomass containing anti-VLY scFv was homogenised in 100 ml (ratio 1:10, biomass:buffer) of 0.1 M Tris-HCl buffer (pH 7.0) with 5 mM EDTA, 0.1% lysozyme, 0.1% Triton X-100, 1 mM phenylmethylsulphonylfluoride (PMSF) and 2-mercaptoethanol. The homogenizate was stirred for 30 min at room temperature (RT), sonicated and centrifuged for 25 min at 24,500 g. The pellet was washed twice with 100 ml of 1 M NaCl containing 0.1% Tween 80 and then with 100 ml of distilled water. After each washing cycle, the suspension was centrifuged for 25 min at 24,500 g. The pellet containing inclusion bodies after the last washing cycle was solubilised in 10 mM Tris-HCl (pH 7.0) containing 7 M guanidine hydrochloride (GuHCl). The suspension was stirred overnight at 4°C, centrifuged for 25 min at 40,000 g and the supernatant was diluted with 10 mM Tris-HCl buffer (pH 7.0) containing 6 M GuHCl to the final protein concentration of 1 mg/ml. The renaturation of anti-VLY scFvs was performed by adding CuSO_4 _solution to the final concentration of 20 μM and incubation for 1 h at RT. The reaction was stopped by adding EDTA solution to the final concentration of 10 mM. Protein solution was centrifuged for 25 min at 40,000 g and the supernatant was loaded onto the Sephadex G-25 column equilibrated with 25 mM Tris-HCl buffer (pH 8.0) containing 0.25 M Na_2_SO_4_. Fractions with the target protein were collected, pooled and loaded onto the Ni (II) NTA column (Qiagen, Hilden, Germany) (loading buffer: 25 mM Tris-HCl, pH 8.0). The elution was performed with the same buffer supplemented with 1 M imidazole. Fractions containing purified anti-VLY scFv were collected, dialyzed against buffer containing 20 mM Tris-HCl (pH 8.0), 50 mM NaCl and sterile filtered for long term storage.

Aliquots of purified scFv (8 μg) were loaded onto TSK-gel G2000 SWXL column (7.8 × 300 mm) (Tosoh Bioscience, Tokyo, Japan). Proteins were eluted with 50 mM phosphate buffer (pH 7.2) supplemented with 0.15 M NaCl at a flow rate of 0.5 ml/min, and detected by UV absorbance at 215 and 280 nm.

The antigen-binding activity of scFv were analysed by an indirect ELISA as described below.

### Determination of the antigen-binding activity of scFvs by an indirect ELISA

Microtiter plates (Nunc MaxiSorp, Nunc, Rosikilda, Denmark) were coated with recombinant purified VLY [30] by adding 100 μl of VLY solution (5 μg/ml) in coating buffer (50 mM Na-carbonate, pH 9.5) and incubation overnight at 4°C. The plates were blocked for 1 h at RT with 2% bovine serum albumin (BSA) in phosphate-buffered saline (PBS) and then incubated with purified scFv for 1 h at RT. After washing, the plates were incubated for 1 h at RT with anti-His5 MAb (Qiagen, Hilden, Germany) diluted 1:1000 in PBS with 0.1% Tween 20 (PBST) and then incubated for 1 h with goat anti-mouse IgG labelled with horse-radish peroxidase (HRP) (BioRad, Richmond, CA, USA) diluted 1:2000 in PBST. After washing, the enzymatic reaction was developed with TMB substrate (Sigma-Aldrich, St. Louis, USA) and stopped by adding 1 M H_2_SO_4_. The optical density was measured at 450 nm (OD_450_) in a microtiter plate reader (Tecan, Groedig, Austria). The apparent dissociation constant (K_d_) of the scFvs was determined by an indirect ELISA as previously described for full-length MAbs [30]. Briefly, scFvs were incubated in VLY-coated plates at concentrations ranging from 50 μg/ml to 20 ng/ml. The plates were incubated with anti-His5 MAb (Qiagen) and HRP-labelled anti-mouse IgG (BioRad) and then developed with TMB substrate. The apparent K_d _was calculated from a titration curve and defined as a molar concentration of scFv corresponding to the mid-point between maximum OD_450 _value and the background.

### Hemolytic *in vitro *assay using human erythrocytes

The use of human erythrocytes from healthy adult volunteer following written informed consent was approved by the Council of the Institute of Biotechnology (Protocol of 30/03/2010, no. 3). *In vitro *neutralization of VLY hemolytic activity by anti-VLY scFv was analysed as described previously [30]. Briefly, blood was collected by a venipuncture from healthy adult volunteer and anticoagulated with EDTA. Erythrocytes obtained from human blood were isolated by centrifugation, washed and resuspended in sterile PBS. Recombinant VLY (5 ng/ml) was preincubated for 30 min at RT with serial dilutions of anti-VLY scFv and added to 1 ml of 1% erythrocyte suspension in PBS. After 15 min of incubation at RT the cells were pelleted by centrifugation and the released hemoglobin was measured at 415 nm in a microplate reader (Tecan). As a positive control for the neutralization assay, VLY pre-incubated with the full-length murine MAb 9B4 was included. As a negative control, the erythrocyte suspension was incubated with VLY alone. The IC_50 _for the scFvs was defined as the concentration of the respective recombinant antibody (M) required for reducing VLY hemolytic activity by 50%.

### Cytotoxicity assay using HeLa cell line

Adherent human cervical epithelial HeLa cells (ATCC Cat. No. CCL-2) were cultivated in RPMI-1640 growth medium (Biochrom, Berlin, Germany) supplemented with 10% fetal bovine serum (Biochrom) and antibiotics. The cells were grown at 37°C and 5% CO_2 _in 96-well plates to approx. 70% confluence. After removing growth medium, the cell monolayer was rinsed twice with serum-free RPMI-1640 medium and then serum-free RPMI-1640 medium was added to the cells (50 μl/well). Recombinant VLY was diluted in serum-free RPMI media and preincubated with serial dilutions of anti-VLY scFv for 30 min at RT. The concentration of VLY in each incubation mixture was 6 μg/ml, the concentrations of anti-VLY scFv ranged from 1.7 mg/ml to 4 μg/ml. Fifty-microliters of each mixture were added to the wells with HeLa cells and the plates were incubated for 1 h at 37°C and 5% CO_2_. Thus, after adding the incubation mixture (50 μl/well) to the culture of HeLa cells (50 μl/well) the final concentration of VLY in each well was 3 μg/ml. As a negative control, recombinant VLY at the final concentration of 3 μg/ml was used. As a positive control, VLY pre-incubated with the full-length neutralizing monoclonal antibody 9B4 (50 μg/ml) was used. Each sample was run in triplicates. After incubation, cell viability was determined by colorimetric assay using 3-(4,5-dimethylthiazol-2-yl)-5-(3-carboxymethoxyphenyl)-2-(4-sulfophenyl)-2H-tetrazolium (MTS) staining. Twenty-microliters of ready-to-use MTS solution (Promega, Madison, USA) were added to the wells and the plates were incubated for 2 h at 37°C and 5% CO_2_. The OD was measured at 490/630 nm wave length in a microplate reader (Tecan). Cell viability was also assessed microscopically at magnifications 40× and 100× using microscope Olympus IX-70 (Olympus, Japan).

### Analysis of the stability of anti-VLY scFvs *in vitro*

In order to evaluate the stability of anti-VLY scFvs, the purified proteins were stored in 20 mM Tris-HCl (pH 8.0), 50 mM NaCl solution at 3 different concentrations: 0.1, 0.31 and 0.65 mg/ml. Protein samples were stored at 4°C for 12 months. The stability of the samples stored at 4°C was analysed at different time points (1, 3, 6, 9, 12 months). Formation of degradation products was evaluated by SDS-PAGE. The neutralizing activity of the stored scFvs was determined by the *in vitro *hemolytic assay.

## Results

### Production of anti-VLY scFvs in *E.coli*

**Figure 1:**
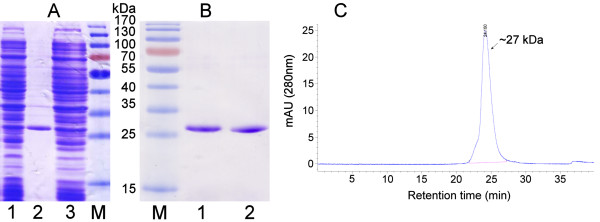
**SDS-PAGE and size-exclusion chromatography of anti-VLY scFv**. (A) Detection of expression of anti-VLY scFv 9B4 in *E. coli *by SDS-PAGE. Lane 1 - soluble fraction of the cell lysate, lane 2 - insoluble fraction of the cell lysate, lane 3 - total cell lysate. (B) SDS-PAGE of purified anti-VLY scFvs derived from hybridoma clones 9B4 (lane 1) and 23A2 (lane 2) by metal-chelate affinity chromatography. Lane M - pre-stained protein molecular weight marker (Thermo Scientific Fermentas). (C) Size-exclusion chromatography of purified anti-VLY scFv 9B4 (variant VL-VH) on a TSK-gel G2000 SWXL column. The column was calibrated with Gel Filtration LMW Calibration Kit (GE Healthcare, Uppsala, Sweden).

The variable regions of the neutralizing anti-VLY MAbs 9B4 and 23A2 were cloned from the respective hybridoma cell lines [30]. The cDNA sequences encoding VH and VL regions of MAbs 9B4 and 23A2 are deposited in GenBank under accession numbers JF951747, JF951748, JF951745 and JF951746. For each hybridoma clone, two variants of anti-VLY scFvs consisting of either VL-VH or VH-VL linked with 20 aa-long linker sequence (G_4_S)_4 _were constructed. The expression plasmids based on pET28a(+) vector bearing anti-VLY scFv encoding genes were transformed into *E.coli *BL21(DE3) strain. The analysis of soluble and insoluble fractions of transformed *E.coli *cells by SDS-PAGE demonstrated that over 90% of the recombinant proteins of interest are found in the insoluble fraction (Figure 1A). The expression levels of different anti-VLY scFvs determined by SDS-PAGE were similar and ranged from 20 to 30% of total cell protein (Figure 1A, Table 1).

**Table 1:** Expression level and stability of anti-VLY scFvs

Anti-VLY scFv	Expression level, % of total cell protein	Oligomeric state	Activity (%) after 12 months storage at 4°C
scFv 9B4 (VL-VH)	20-30	monomeric	100
scFv 9B4 (VH-VL)	20-30	monomeric	100
scFv 23A2 (VL-VH)	20-30	monomeric	75
scFv 23A2 (VH-VL)	20-30	monomeric	75

The N-terminally hexahistidine-tagged anti-VLY scFvs representing 27.5 kDa proteins were purified from inclusion bodies by renaturation and subsequent application of metal-chelate affinity chromatography. Preparations of purified anti-VLY scFv verified by SDS-PAGE under reducing conditions represented essentially homogenous scFvs (Figure 1B). Size exclusion chromatography of anti-VLY scFvs revealed one major peak with an apparent molecular weight corresponding to monomeric state of scFv (Figure 1C, Table 1). No fractions corresponding to the oligomeric forms of scFv were eluted from the column. This indicated the low tendency of generated anti-VLY scFvs to form multimeric structures. Thus, after renaturation and purification steps four variants of monovalent scFvs derived from the respective hybridomas 9B4 and 23A2 were obtained.

### Neutralization of VLY hemolytic activity by anti-VLY scFvs

**Figure 2:**
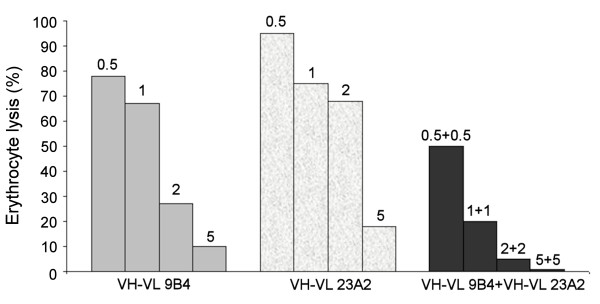
**Inhibition of VLY-mediated hemolysis of human erythrocytes by anti-VLY scFvs**. Erythrocyte lysis was evaluated after incubation of VLY either with a separate anti-VLY scFv or the mixture of two anti-VLY scFvs (VH-VL variant) derived from hybridoma clones 9B4 and 23A2. The amounts of anti-VLY scFvs (μg) added to the fixed amount of VLY (5 ng per sample) are indicated on the top of each column.

The ability of anti-VLY scFvs to inhibit the hemolytic activity of VLY was investigated using human erythrocytes. In our previous study the MAbs 9B4 and 23A2 have been shown to neutralize the activity of VLY and prevent the lysis of human erythrocytes *in vitro*. The MAb 23A2 was shown to exhibit fair neutralizing activity (IC_50 _= 4.8 × 10^-10 ^M) while the MAb 9B4 showed the more potent neutralizing activity (IC_50 _= 6.7 × 10^-11 ^M) [30]. We have analysed the neutralizing potency of different anti-VLY scFv constructs in comparison with that of full-length MAbs 9B4 and 23A2. All purified anti-VLY scFvs were able to neutralize the hemolytic activity of VLY (Figure 2, Table 2). The IC_50 _values calculated from concentration-dependent curves ranged from 3.7 × 10^-8 ^to 1.5 × 10^-7 ^M. The anti-VLY scFvs, both VL-VH and VH-VL variants, derived from the parental hybridoma clone 9B4 exhibited stronger neutralizing activity (IC_50 _= 3.7 × 10^-8 ^M and 5.1 × 10^-8 ^M, respectively) than anti-VLY scFvs derived from hybridoma clone 23A2 (IC_50 _= 1.1 × 10^-7 ^M and 1.5 × 10^-7 ^M, respectively). The extent of the neutralizing potency of different scFvs was in agreement with that of their parental full-length MAbs (Table 2). The antigen-binding activities of purified scFvs were investigated by an indirect ELISA. All scFv variants recognized recombinant VLY immobilized on the plate and showed moderate binding affinities with the apparent K_d _1.1 × 10^-8 ^to 9.8 × 10^-8 ^M that was also in line with the affinities of parental full-length MAbs (Table 2). No VLY-neutralizing activity was observed when a control scFv derived from the non-neutralizing hybridoma clone 10G5 was used (data not shown). A mixture of equal amounts of two different anti-VLY scFvs showed substantially higher neutralizing potency on the hemolytic activity of VLY as compared to single scFv (Figure 2). The additive action of two anti-VLY scFvs on the neutralization of VLY activity indicates the bivalent manner of the mixture of anti-VLY scFvs.

**Table 2:** Neutralizing activity and affinity of anti-VLY scFvs and the respective MAbs

Anti VLY scFv and MAb	IC_50_, M	K_d_, M
scFv 9B4 (VL-VH)	3.7 × 10^-8^	1.1 × 10^-8^
scFv 9B4 (VH-VL)	5.1 × 10^-8^	1.5 × 10^-8^
scFv 23A2 (VL-VH)	1.1 × 10^-7^	9.2 × 10^-8^
scFv 23A2 (VH-VL)	1.5 × 10^-7^	9.8 × 10^-8^
MAb 9B4	6.7 × 10^-11^	2.9 × 10^-10^
MAb 23A2	4.8 × 10^-10^	1.2 × 10^-10^

### Neutralization of VLY cytolytic activity by anti-VLY scFvs

**Figure 3:**
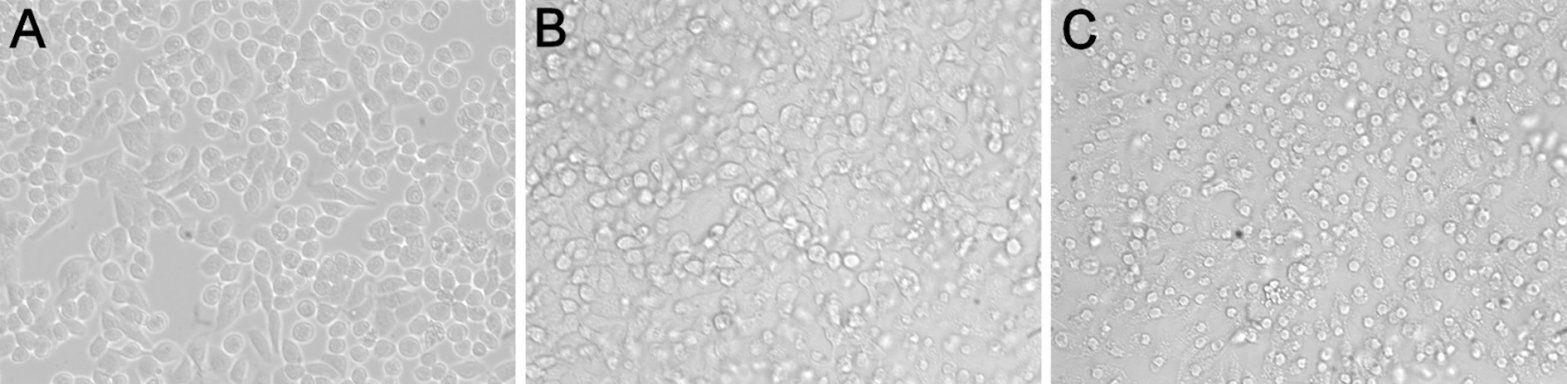
**HeLa cell culture after 10 min of incubation with different concentrations of VLY: 0.03 μg/ml (A), 0.3 μg/ml (B), 3 μg/ml (C)**. The cells were observed by microscope Olympus IX-70 at magnification 40×.

**Figure 4:**
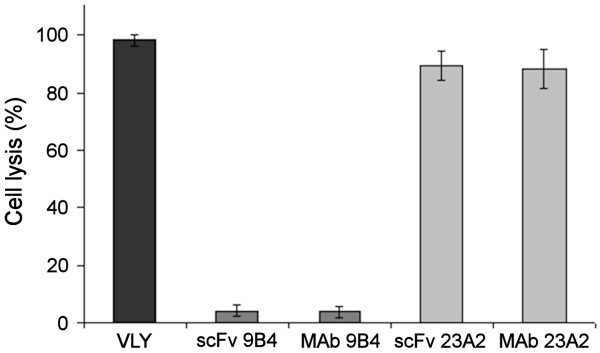
**Inhibition of VLY-mediated cytolysis of HeLa cells by anti-VLY scFvs**. HeLa cells were exposed to VLY alone (3 μg/ml) or VLY (3 μg/ml) preincubated either with scFvs (500 μg/ml) or full-length MAbs (50 μg/ml). Cell viability was determined by colorimetric assay using MTS staining. Mean OD_490 _values (± 2SD) were calculated from triplicates. The neutralizing activity of each anti-VLY scFv variant is expressed in % of cytolysis. Complete cytolysis induced by VLY alone is set 100%.

The neutralizing potency of anti-VLY scFvs was assayed using human cervical epithelial (HeLa) cells. Lysis of vaginal epithelial cells by VLY is considered to be a key step in progression of bacterial vaginosis and predispose to further complications associated with the disease [3,20]. Thus, inhibition of cytolytic activity of VLY may have important physiologic relevance. In a first step the concentration of VLY required for a complete lysis of HeLa cells as well as the dynamics of cytolysis were investigated. For this purpose, the cells were incubated with increasing VLY concentrations (from 1 ng/ml to 100 μg/ml) and observed microscopically at different time points (10-60 min). In parallel, the viability of HeLa cells was measured by colorimetric MTS assay. It was determined that 2.5 μg/ml of VLY is sufficient to induce complete lysis of HeLa cells (Figure 3C). The cytolytic effect of VLY on HeLa cell culture was observed immediately (10 min) after VLY adding. Thus, it was demonstrated that the quantities of VLY required to induce the lysis of HeLa cells are higher than that required to induce the lysis of human erythrocytes (2.5 μg/ml and 5 ng/ml, respectively). The ability of scFvs and the respective full-length MAbs to inhibit VLY-mediated cytolysis of HeLa cells was evaluated by colorimetric MTS assay. It was determined that 500 μg/ml of scFv and 50 μg/ml of full-length antibody 9B4 induced complete neutralization of VLY-mediated cytolysis in HeLa culture (Figure 4). In contrast, the scFv derived from hybridoma 23A2 did not inhibit VLY cytolytic activity even at the highest concentration used (1 mg/ml), which is explained by its lower affinity and neutralizing potency (Table 2). In line with these data, the full-length MAb 23A2 was not able to inhibit VLY cytolytic activity at concentration of 50 μg/ml and higher (Figure 4).

### Stability of scFvs during long-term storage

**Figure 5:**
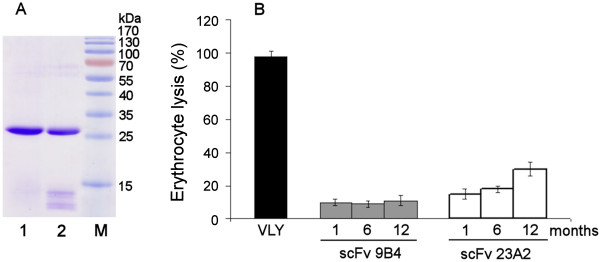
**Stability of anti-VLY scFvs during long-term storage**. (A) Analysis of anti-VLY scFvs stored at the concentration of 0.65 mg/ml for 12 months by SDS-PAGE under reducing conditions. Lane 1 - 9B4-derived scFv (variant VH-VL); lane 2 - 23A2-derived scFv (variant VH-VL). Lane M - pre-stained protein molecular weight marker (Thermo Scientific Fermentas). (B) Testing of anti-VLY scFvs stored at the concentration of 0.65 mg/ml for 1, 6 and 12 months for the ability to neutralize VLY-mediated lysis of human erythrocytes.

The activity of anti-VLY scFvs stored for 12 months at 4°C was tested by determination of their ability to neutralize VLY-mediated lysis of human erythrocytes *in vitro*. The appearance of possible degradation products was analysed by SDS-PAGE. The protein samples stored at three different concentrations were analyzed at different time points. The SDS-PAGE under denaturing conditions did not reveal any degradation products of anti-VLY scFv (VH-VL and VL-VH variants) derived from hybridoma clone 9B4 stored for 12 months at 4°C even at the highest protein concentration (Figure 5A). However, both variants of scFv derived from hybridoma clone 23A2 showed minor degradation products when stored for more that 6 months (Figure 5A). The neutralizing potency of VLY hemolytic activity by 23A2-derived scFv declined during storage, while the neutralizing activity of 9B4-derived scFvs remained unchanged (Figure 5B, Table 1). Thus, VH-VL and VL-VH variants of 9B4-derived scFv were more stable during long-term storage at 4°C as compared to 23A2-derived scFvs.

## Discussion

The increasing number of antibiotic resistant bacteria and the severity of infections upgrade the interest in antibody therapy against toxins associated with the development of disease. The high recurrence rate or persistence of infection, inappropriate or controversial treatment with antibiotics, extreme toxicity and potency of the toxins released from killed bacteria are suggested to be the cases applicable for passive immunotherapy [26,29,33]. Recombinant scFvs either derived from hybridoma cells or produced by phage display technique remain an attractive alternative to full-length antibodies because of easier and less costly manufacture, opportunity of genetic engineering, tissue penetration and short-circulating half-lives. It was demonstrated by *in vitro *and *in vivo *assays that recombinant antibodies neutralize the activity of toxins that are highly diverse in nature and mode of action [23-29]. Hybridoma-derived scFvs were shown to neutralize the cytolytic activity of pneumolysin from *Streptococcus pneumonia *[23]. Recently, human scFvs derived from a phage display library were developed that neutralize the cytotoxicity of Shiga toxins produced by enterohemorragic *E. coli *bacteria [29]. The efficacy of single domain antibodies (VHH) to neutralize *Clostridium difficile *toxin A was demonstrated by the *in vitro *assay [28]. The ability to neutralize botulinum neurotoxin in mice was demonstrated using chimeric antibody generated by fusion of hybridoma-derived scFv with human Fc domain [26].

The secreted toxin VLY is known to be the major virulence factor of *G. vaginalis *[16,17]. Toxic effect of VLY is based on its ability to form pores in the cell membrane causing cell lysis [16,17,19]. Recently, novel murine MAbs 9B4 and 23A2 against VLY were developed and characterized as highly efficient in neutralizing VLY activity *in vitro *[30]. In the current study, we generated and characterized four functionally active anti-VLY scFvs derived from hybridomas 9B4 and 23A2. Anti-VLY scFvs with hexahistidine residues at the N-terminus were expressed in *E. coli *and recovered from the inclusion bodies with subsequent application of metal-chelate affinity chromatography.

Purified anti-VLY scFvs inhibited VLY-mediated cytolysis with different efficiency that was in line with the affinity and neutralizing potency of the respective parental MAbs. The anti-VLY scFvs derived from hybridoma 9B4 exhibited high VLY-neutralizing activity both on human erythrocytes and cervical epithelial HeLa cells. Moreover, the 9B4-derived scFvs were shown to be highly stable during long-term storage at 4°C. Therefore, this construct may be considered as a promising candidate for developing novel therapeutic tools to neutralize cytotoxic effects of VLY.

Interestingly, VH-VL and VL-VH variants of scFv showed similar affinity and neutralizing potency. This suggests that the orientation of variable fragments in the construct did not influence dramatically the folding and subsequently the antigen-binding capacity of the scFv. However, recombinant anti-VLY scFvs did not retain all the properties of the respective parental MAbs. The decreased neutralizing potency and reduction in affinity of scFvs as compared to full-length MAbs can be explained by monovalent binding properties, as purified anti-VLY scFvs were found predominantly in a monomeric form. The avidity could be increased by generation of recombinant antibodies with multivalent binding capacity using the scFv multimerization approach or generation of a fusion with the whole Fc fragment of human IgG [26,34,35]. The scFvs described in the current study is the first example of recombinant single-chain monovalent antibodies with VLY-neutralizing activity produced in prokaryote expression system.

## Conclusions

We have expressed in *E.coli *hybridoma-derived scFvs with VLY-binding activity. Anti-VLY scFvs inhibited VLY-mediated lysis of human erythrocytes *in vitro *and human cervical epithelial HeLa cells. The monovalent scFvs showed reduced affinity and neutralizing potency as compared to the respective parental MAbs. The loss of avidity could be restored by generating scFv constructs with multivalent binding properties. *G. vaginalis *caused infections continue to be a world-wide problem, therefore neutralizing recombinant antibodies may provide novel therapeutic tools useful in the treatment of BV and other diseases caused by *G. vaginalis*.

## Competing interests

The authors declare that they have no competing interests.

## Authors' contributions

MP carried out all gene engineering procedures, analysed neutralizing activity of scFvs and drafted the manuscript. EM purified recombinant VLY and scFvs. RL carried out the immunoassay and cytolytic assay. GZ selected the primers for cloning and helped to interpret experimental data. AZ participated in the design of the study and revised the manuscript. All authors read and approved the final manuscript.
